# Self-Tuning Threshold Method for Real-Time Gait Phase Detection Based on Ground Contact Forces Using FSRs

**DOI:** 10.3390/s18020481

**Published:** 2018-02-06

**Authors:** Jing Tang, Jianbin Zheng, Yang Wang, Lie Yu, Enqi Zhan, Qiuzhi Song

**Affiliations:** 1 School of Information Engineering, Wuhan University of Technology, Wuhan 430070, China; mimitang85119@163.com (J.T.); jbzheng@whut.edu.cn (J.Z.); eqzhan@whut.edu.cn (E.Z.); 2 Key Laboratory of Fiber Optic Sensing Technology and Information Processing, Ministry of Education, Wuhan University of Technology, Wuhan 430070, China; 3 School of Electronic and Electrical Engineering, Wuhan Textile University, Hongshan District, Wuhan 430070, China; lyu@wtu.edu.cn; 4 School of Electromechanical, Beijing Institute of Technology, Beijing 100081, China; qzhsong@bit.edu.cn

**Keywords:** adaptability, force sensitive resistors, self-tuning triple threshold algorithm

## Abstract

This paper presents a novel methodology for detecting the gait phase of human walking on level ground. The previous threshold method (TM) sets a threshold to divide the ground contact forces (GCFs) into on-ground and off-ground states. However, the previous methods for gait phase detection demonstrate no adaptability to different people and different walking speeds. Therefore, this paper presents a self-tuning triple threshold algorithm (STTTA) that calculates adjustable thresholds to adapt to human walking. Two force sensitive resistors (FSRs) were placed on the ball and heel to measure GCFs. Three thresholds (i.e., high-threshold, middle-threshold andlow-threshold) were used to search out the maximum and minimum GCFs for the self-adjustments of thresholds. The high-threshold was the main threshold used to divide the GCFs into on-ground and off-ground statuses. Then, the gait phases were obtained through the gait phase detection algorithm (GPDA), which provides the rules that determine calculations for STTTA. Finally, the STTTA reliability is determined by comparing the results between STTTA and Mariani method referenced as the timing analysis module (TAM) and Lopez–Meyer methods. Experimental results show that the proposed method can be used to detect gait phases in real time and obtain high reliability when compared with the previous methods in the literature. In addition, the proposed method exhibits strong adaptability to different wearers walking at different walking speeds.

## 1. Introduction

From a medical and industrial perspective, wearable devices have evolved and continue to develop in terms of providing assistance to humans [1]. Gait analysis through wearable devices is an extensive area in the field of biomechanics that provides knowledge in terms of identifying pathologies, evaluating athletes’ performance, design of sports products and rehabilitation engineering [2]. Generally, the wearable devices used for gait analysis are designed by equipping various sensors on it.

Thus far, different kinds of sensor types, including force sensitive resistors (FSRs) [3,4,5], air pressure sensors [6], inertial sensors [7,8,9,10,11,12,13,14], inclinometers [15], foot switches [16], and electromyography (EMG) sensors [17], are readily available in the industry and can be applied to gait analysis. Yan et al. [18] estimated the gait events through a sensory apparatus, which combine three subsystems such as a primary phase estimator, a desired gait event detector and a phase error compensator. This method is sensitive to the tuning of the adaptive oscillators parameters. However, different parameters may change the results. In the literature, several works [11,12] have detected the gait phase using the inertial sensors that could measure the body segment orientations and joint angles. Bejarano et al. [13] proposed an adaptive algorithm based on the inertial and magnetic sensors to detect the gait events. However, the inertial sensor is sensitive to temperature, shock and magnetic disturbances, which would result in the misdetections of gait phases. In order to overcome these limitations, Muller et al. [14] proposed a novel online gait phase detection algorithm, which can be used indoor and in the presence of magnetic disturbances. However, the delays of the heel off and initial contact are considerably high. Nevertheless, an alternative solution to inertial sensors is FSRs [16]. As described by Catalfamo et al. [19], force platforms, such as FSRs, represent the gold standard method for gait analysis. Yu et al. [20] used proportion an adaptive method, which calculates the sums and proportions of ground contact forces (GCFs). However, one parameter is affected by the attachment of the shoe to the foot, which would affect the reliability of the whole system. Additionally, force platforms measure the GCFs to detect gait phases, and each gait phase has a unique GCF pattern [7].

As reported by Smith et al. [21], 80% of errors in gait phase detection using FSRs was due to the setting of threshold value. In order to set appropriate thresholds for gait phase detection, a number of researchers [4,5,19] had presented their methods. Mariani et al. [4] defined 5% body weight as a threshold. However, different subjects had different body weights such that different thresholds should be set for different subjects. In addition, no matter how slowly or fast the subject walked, only one threshold was set for the same subject in all experiments. As a result, this method of using body weight percentage as a threshold was not adaptable to different subjects and different walking speeds (i.e., slow and fast). Lopez–Meyer et al. [5] and Catalfamo et al. [19] used the maximum and minimum GCFs of gait cycles to compute the threshold. However, the use of the maximum and minimum GCFs for the threshold computation could not detect gait phases in real time because the maximum and minimum GCFs were obtained in data post-processing.

The main purpose of this study is to develop a gait phase detection method, which can detect gait phases in real time during different walking trials. Therefore, we propose a self-tuning triple-threshold algorithm (STTTA), which obtains the maximum and minimum GCFs of the present gait cycle to calculate adjustable thresholds. In addition, the acquirement of the maximum and minimum GCFs, and the calculations of adjustable thresholds are completed in the walking process. This paper also proposes a gait phase detection algorithm (GPDA), which provides the rules that determine calculations for STTTA. In order to evaluate the reliability of STTTA, three previous methods in the literature are introduced to obtain comparative results.

We hypothesize that the proposed STTTA can gain high and stable reliabilities for different subjects walking at different speeds. To better test the adaptability, the same initial threshold values are set for all subjects in all experiments.

## 2. Methods

### 2.1. Subjects

This study included fourteen males and ten females (age = 24.5 ± 2.0 years, weight = 67.3 ± 8.8 kg; mean ± SD) with no history of foot diseases. All of them volunteered to participate in our experiments. The participants walked on the treadmill for 30 s duration at designated constant speeds of 2 km/h, 3 km/h, 4 km/h, 5 km/h and 6 km/h, respectively.

### 2.2. Instrumentation and Data Processing

**Figure 1:**
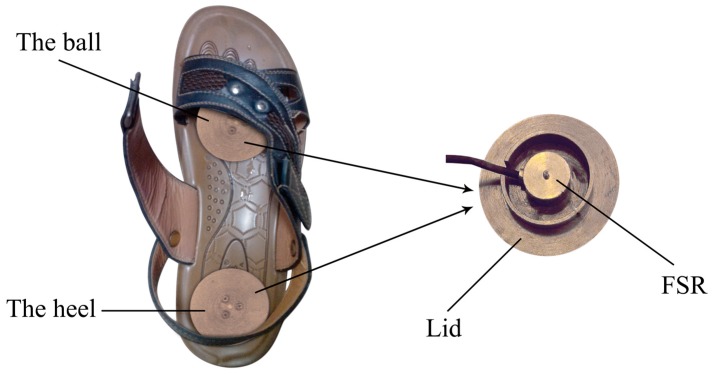
FSRs placed inside one shoe with one in the ball and the other in the heel. A lid is made to enlarge the press area.

As shown in Figure 1, the sensor units comprised two FSRs (LOSON LSH-10, LOSON Instrumetation, Nankin, China), which were located in the sole of the ball and the sole of the heel. The FSRs collected GCF data at a frequency of 2000 Hz with a high resolution of 16 bits AD converter using an ARM11 computer (S3C6410). The measuring range of each FSR was 0–200 kg. The accuracy (including linearity and repeatability) of each FSR was ±0.5% full scale (FS). Standard load cells (5 kg, 10 kg, 20 kg, 25 kg, 50 kg, 100 kg and 200 kg) were used to calibrate the FSRs. As the FSRs output a weak micro-voltage signal, an amplification circuit was equipped. Because of this circuit, the output signal of each FSR was amplified to 0–5 V, which correlated with the measured mass of 0–200 kg.

After data acquisition, the GCF data from FSRs were filtered by second order Butterworth low pass filter with a cut-off frequency of 10 Hz. The results of the gait phase detection were processed in Matlab (version2012, MathWorks, MA, USA) using the proposed method.

### 2.3. Previous Methods

In previous, the GCFs measured by an FSR can be divided into on-ground and off-ground statuses through setting a threshold *T*:

(1) $$G=\left\{ \begin{array}{l} on-ground,\;F\geq T\\ off-ground,\;F<T \end{array}\right.,$$

where *F* is the GCF from the ball or heel, and Tis the threshold. *G* is the result that “on-ground” status means “FSR pressed” and “off-ground” status means “FSR not pressed”.

In our experiments, three previous ways were applied to the threshold computations. To be specific, one way was to define body weight percentage as a threshold, which had been affirmed to detect gait phase in real time. The other two ways were to use the maximum and minimum GCFs of gait cycles for threshold calculation. Meanwhile, the two ways carried out data post-processing to calculate an appropriate threshold, and were finally chosen as reference methods.

#### 2.3.1. Mariani Method

Mariani et al. [4] defined 5% of body weight to compute the threshold *T*:

*T* = 0.05∙mg, (2)

where m is the subject’s mass and g is the acceleration of gravity.

#### 2.3.2. Timing analysis module Method

Catalfamo et al. [19] employed a Timing analysis module (TAM) software (version5.24, Tekscan, South Boston, MA, USA) as their reference method. After data acquisition of each experiment, the maximum and minimum GCFs (i.e., *T*_max_ and *T*_min_, respectively) were searched out for threshold calculation:

(3) $$T=T_{\min}+(T_{\max}-T_{\min})\times \frac{10}{100}.$$

Additionally, *T* needed to be calculated severally for each set of FSR in each experiment.

#### 2.3.3. Lopez–Meyer Method

Lopez–Meyer et al. [5] used the average value of the maximum GCFs and the average value of the minimum GCFs to compute the threshold *T*. For each experiment, the threshold computation needed all the local maximum GCFs and local minimum GCFs (i.e., *T*_max_(*i*) and *T*_min_(*j*), respectively) of gait cycles. In a complete gait cycle, there were only one *T*_max_ and only one *T*_min_ for each set of FSR. In one experiment, there were many gait cycles. For one set of FSR, there were *k T*_max_ and *l T*_min_ in one experiment. However, *k* did not equal *l* as there might exist an incomplete gait cycle in the experiment:

(4) $$T_{MAX}=\frac{1}{k}\sum_{i=1}^{k}T_{\max}(i),$$

(5) $$T_{MIN}=\frac{1}{l}\sum_{j=1}^{l}T_{\min}(j),$$

(6) $$T=T_{MIN}+\alpha (T_{MAX}-T_{MIN}),$$

where *α* was a proportional factor for the threshold adjustment to compensate for interindividual variability in pressure levels. In our experiments, the selection of proportional factor was made that α was set to 0.084.

### 2.4. Self-Tuning Triple-Threshold Algorithm

With respect to the Mariani method, different thresholds should be set for different subjects, and the same threshold is set for one subject in five experiments with different walking speed each time. The Mariani method is not adaptable to different subjects and different walking speeds. Meanwhile, the TAM and Lopez–Meyer methods both carry out data post-processing to calculate the thresholds and are incapable of real-time application. In order to seek a method to detect gait phase in real time and be adaptable to different walking conditions, we propose a self-tuning triple-threshold algorithm (STTTA) that uses three thresholds to obtain the maximum and minimum GCFs for threshold computations.

#### 2.4.1. Setting of Three Thresholds

**Figure 2:**
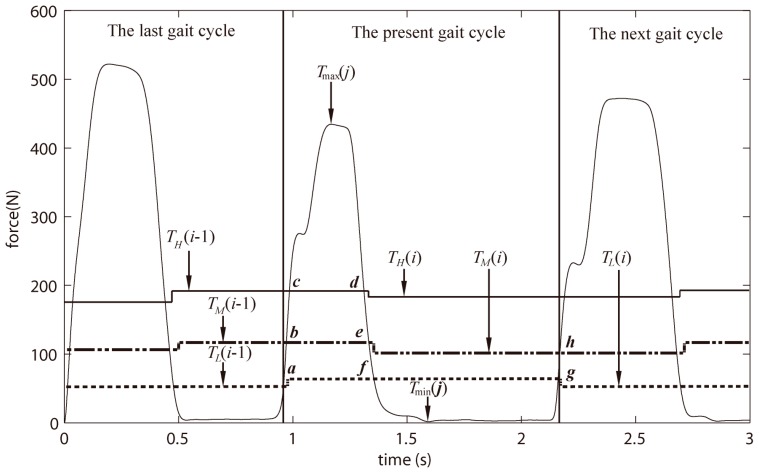
Three thresholds used for the GCFs processing.

In this section, the magnitudes of three thresholds are amplified. Figure 2 demonstrates the GCFs processing through three thresholds, including high-threshold, middle-threshold and low-threshold (i.e., *T_H_*, *T_M_* and *T_L_*, respectively). Specifically, *T_H_* is utilized to search the maximum GCF (i.e., *T*_max_(*j*)) of the present gait cycle, and chosen as the main threshold that divides the GCFs into on-ground and off-ground statuses. *T_M_* is used to search time points (points b, e and h shown in Figure 2) for the adjustments of *T_H_* and *T_L_*. Meanwhile, *T_L_* is employed to search the minimum GCF (i.e., *T*_min_(*j*)) of the present gait cycle, and the time point (point f) for the adjustments of *T_M_*.

At the present gait cycle, the three thresholds are newly calculated for the next gait cycle. The three thresholds (i.e., *T_H_*(*i* − 1), *T_M_*(*i* − 1) and *T_L_*(*i* − 1)), by which the GCFs are processed at the present gait cycle, have been calculated at the last gait cycle.

As Figure 2 shows, the present gait cycle starts at point a and ends at point g. Point a is the end point of the last gait cycle, and also the start point of the present gait cycle. Point b is used to calculate the *T_L_*(*i* − 1). Points c and d make a region [c, d]. When the GCF (*F*(*k*)) is larger than *T_H_*(*i* − 1), it goes to region [c, d]. Then, the *T*_max_(*j*) can be searched out as follows:

(7) $$T_{\max}(j)=MAX=\left\{ \begin{array}{l} F(k),\;F(k)\geq T_{H}(i-1)\&F(k-1)<T_{H}(i-1)\\ F(k),\;F(k)>MAX\&F(k)>T_{H}(i-1)\&F(k-1)\geq T_{H}(i-1)\\ MAX,\;F(k)<MAX\&F(k)>T_{H}(i-1)\&F(k-1)>T_{H}(i-1) \end{array}\right.,$$

where *k* is the total number of the collected GCFs from one FSR in each experiment. The numerical range of *k* is 1~60,000 because each experiment lasts 30 s with a sampling frequency of 2000 Hz.

At point e, *T_H_*(*i*) is calculated as a new threshold for the next gait cycle:

(8) $$T_{H}(i)=\beta \cdot (T_{\max}(j)-T_{L}(i-1))+T_{L}(i-1),$$

where *β* is a proportion factor and chosen to be a constant. *i* means that the present gait cycle is the *i*-th step of one subject walking in one experiment, while *j* means that the *T*_max_(*j*) is the *j*-th maximum GCF of the total gait cycles. However, *i* was not necessarily equal to *j* as incomplete gait cycles may have occurred.

At point f, *T_M_*(*i*) is newly calculated for the next gait cycle:

(9) $$T_{M}(i)=\gamma \cdot (T_{\max}(j)-T_{L}(i-1))+T_{L}(i-1),$$

where *γ* is a proportion factor and chosen to be a constant. 

In common with points c and d, the points f and g also make a region [f, g]. The calculation of *T*_min_(*j*) is made in the regions [f, g], which means that the *F*(*k*) is smaller than *T_L_*(*i* − 1):

(10) $$T_{\min}(j)=MIN=\left\{ \begin{array}{l} F(k),\;F(k)\leq T_{L}(i-1)\&F(k-1)>T_{L}(i-1)\\ F(k),\;F(k)<MIN\&F(k)<T_{L}(i-1)\&F(k-1)\leq T_{L}(i-1)\\ MIN,\;F(k)>MIN\&F(k)<T_{L}(i-1)\&F(k-1)<T_{L}(i-1) \end{array}\right..$$

Additionally, point g is the end point of the present gait cycle, and also the start point of the next gait cycle.

However, *T_L_*(*i*) is newly set at point h, as the same to *T_L_*(*i* − 1) calculated at point b:

(11) $$T_{L}(j)=\lambda \cdot T_{\min}(j)+(1-\lambda )\cdot T_{M}(i),$$

where *λ* is a proportion factor and chosen to be a constant.

#### 2.4.2. Setting of Initial Threshold Values

Before the experiments, it is necessary to set initial values for the three thresholds. In this paper, *T_H_*(1), *T_M_*(1) and *T_L_*(1) are the initial values of *T_H_*, *T_M_* and *T_L_*. However, the setting should follow that *T_L_*(1) < *T_M_*(1) < *T_H_*(1).

### 2.5. Gait Phase Detection Algorithm

The gait cycle can be divided into stance-phase and swing-phase. The transitions between gait phases are gait events. As proposed by Pappas et al. [3,22], swing-phase, stance-phase, heel-strike and heel-off are the most common gait phases and gait events for a single foot. For the proposed STTTA, *T_H_* is the main threshold, which divided the GCFs into on-ground and off-ground statuses:

(12) $$G=\left\{ \begin{array}{l} on-ground,\;F\geq T_{H}\\ off-ground,\;F<T_{H} \end{array}\right..$$

As is the same for Equation (1), *F* is the GCF from the ball or heel, and *T_H_* is the threshold. *G* is the result that “on-ground” status means “FSR pressed” and “off-ground” status means “FSR not pressed”.

When the GCFs from the ball and heel are processed from Equations (1) and (12), the gait phases and gait events can be distinguished by following the rules in Table 1.

### 2.6. Reference Methods

In order to obtain the reliability of the proposed STTTA, reference methods should be determined. The TAM and Lopez–Meyer methods carried out data post-processing to calculate an appropriate threshold for each FSR at each walking speed. In addition, the TAM method had been chosen as a reference method to test the reliability of gait events detection. On the other hand, the Lopez–Meyer method had been tested on a shoe-based wearable sensor system, which had been compared with the “GAITRite system”. The Lopez–Meyer method acquired 95% confidence to compare detection results with the GAITRite system. Therefore, in this paper, the TAM and 

Lopez–Meyer methods were both selected as reference methods. Finally, both the STTTA and Mariani methods were compared with the reference methods to obtain their reliabilities.

## 3. Experimental Results

### 3.1. Selection of Coefficients

Before the experiments, the values of *T_H_*(1), *T_M_*(1) and *T_L_*(1) were determined. The value selections of *T_H_*(1), *T_M_*(1) and *T_L_*(1) followed the rules that *T*_min_ < *T_L_*(1) < *T_M_*(1) < *T_H_*(1) < *T*_max_. Based on data analysis, the value range of *T*_min_ was 0–5 N and the value range of *T*_max_ was 300–1300 N. Therefore, the values of *T_H_*(1), *T_M_*(1) and *T_L_*(1) could be selected within a wide range. To evaluate this in our experiments, we randomly chose a set of initial threshold values where *T_L_*(1) = 15 N, *T_M_*(1) = 20 N and *T_H_*(1) = 25 N.

**Figure 3:**
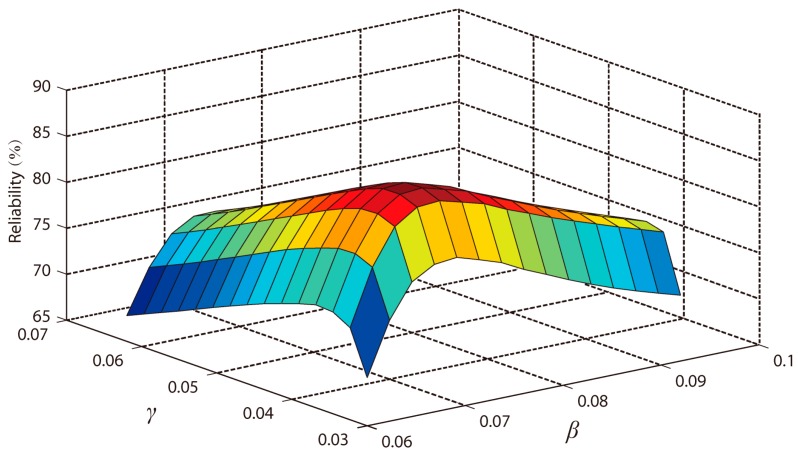
The reliabilities of the proposed STTTA in various values of *β* and *γ*.

After the selection of *T_H_*(1), *T_M_*(1) and *T_L_*(1), the proportion factor β, γ, and λ in Equations (8), (9) and (11) were optimized to determine the highest reliability of STTTA. From Equations (9) and (11), the values of *T_M_* and *T_L_* were affected by each other. As a result, one of γ and λ should be firstly determined such that the value of λ was chosen to be 0.5. Then, using the data from nine subjects as training data, the reliabilities of the proposed STTTA were calculated by using various values of β and γ, which is shown in Figure 3. The selections for β and γ to acquire the highest reliability was made such that β = 0.071 and γ = 0.042.

Finally, the same *T_H_*(1), *T_M_*(1) and *T_L_*(1) were used for all 24 subjects in all experiments. In addition, data from the remaining subjects were processed using the selected β, γ, and λ.

### 3.2. The Results of Gait Phase Detection and Experimental Comparisons

**Figure 4:**
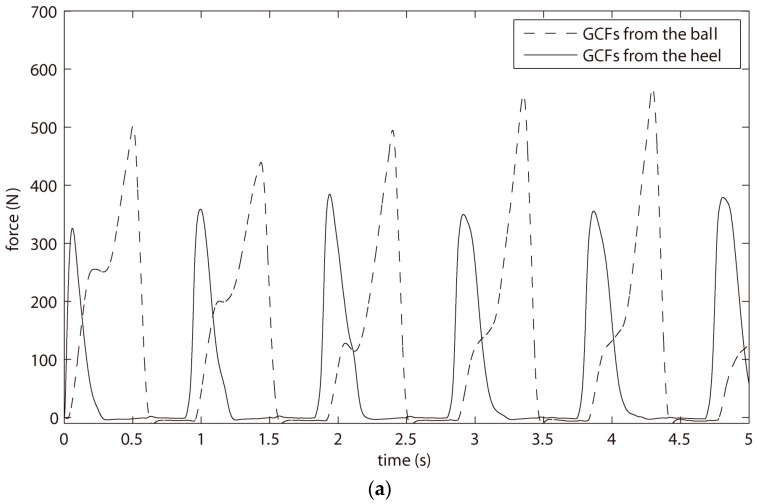

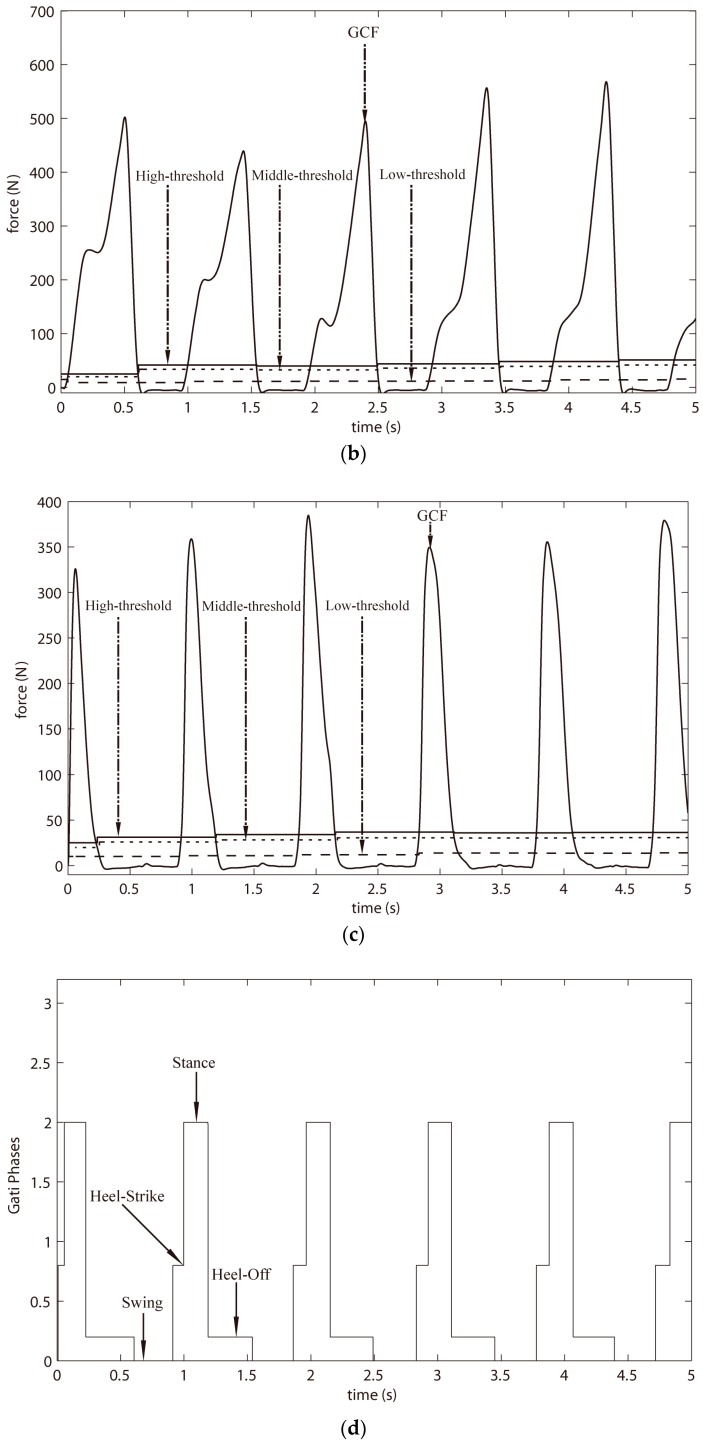

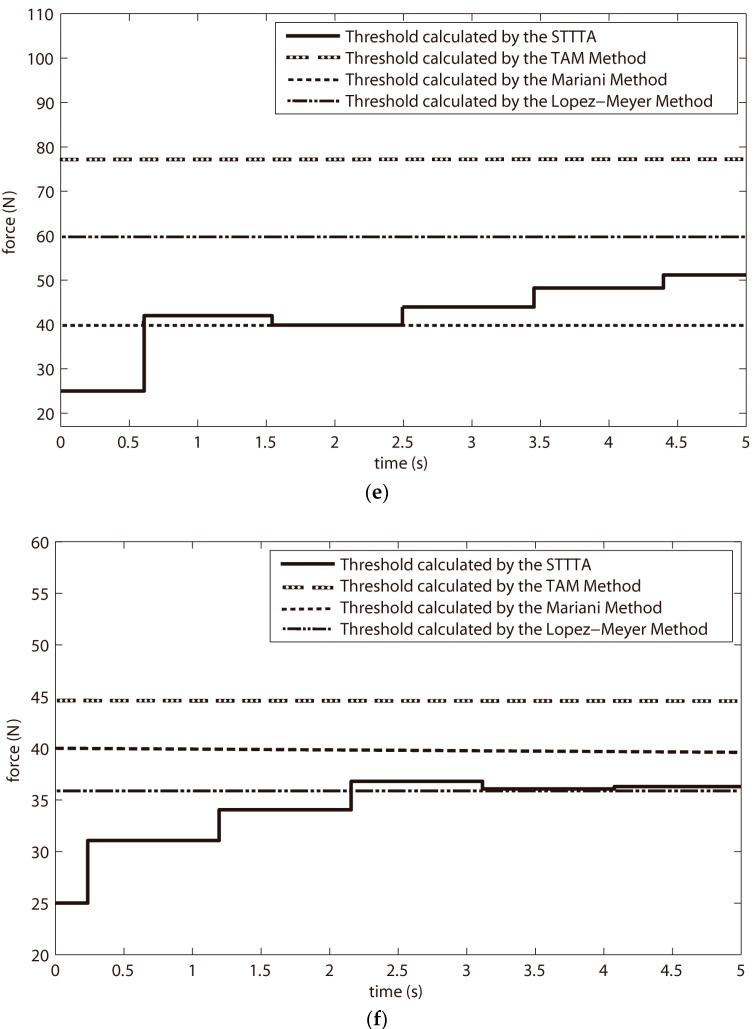
(**a**) the GCFs of heel and ball measured by FSRs; (**b**) three self-tuning thresholds for the processing of GCF from the ball; (**c**) three self-tuning threshold for the processing of GCF from the heel; (**d**) the result of gait phase detection through GPDA; (**e**) thresholds calculated for the ball by four kinds of methods; and (**f**) thresholds calculated for the heel by four kinds of methods.

Figure 4 showed the detection results of the proposed STTTA. In Figure 4a, the GCFs from the ball and heel were displayed. The self-adjustments of three thresholds for the GCFs from the ball and heel is demonstrated in Figure 4b,c. It was qualitatively illustrated in Figure 4d where the gait phases and gait events were detected through the proposed GPDA. In order to clarify the self-adjustment effect, the main threshold T_H_ of the proposed STTTA was compared with the thresholds calculated by the previous methods. As pictured in Figure 4e,f, the amplitudes of the threshold T_H_ were adjustable and adaptable to walking conditions, while constant thresholds were figured out for the previous methods.

### 3.3. Real-Time Application for Gait Phase Detection

After data acquisitions, the data processing included the initial threshold setting, acquirement of the maximum and minimum GCFs, and threshold computations, was made prior to the GPDA. When the threshold computations were achieved, the gait phase detection could be implemented through the GPDA.

In this gait phase detection system, the sampling frequency was set to 2000 Hz. Data acquisitions, data processing and gait phase detection could be all accomplished within one sampling period. The time delay between data acquisition and gait phase detection was less than 0.5 ms. Therefore, the proposed STTTA could be used to detect gait phases in real time.

### 3.4. Adaptability to Different Walking Conditions

Needless to obtain the body weights of all subjects, the proposed STTTA acquired higher average reliabilities than the Mariani method as shown in Table 2. In addition, irrespective of the reference method applied (TAM method or the Lopez–Meyer method), higher reliability was gained for the STTTA compared to the Mariani method. The comparative results affirmed that the proposed STTTA was more reliable than the Mariani method in real-time gait phases detection.

**Figure 5:**
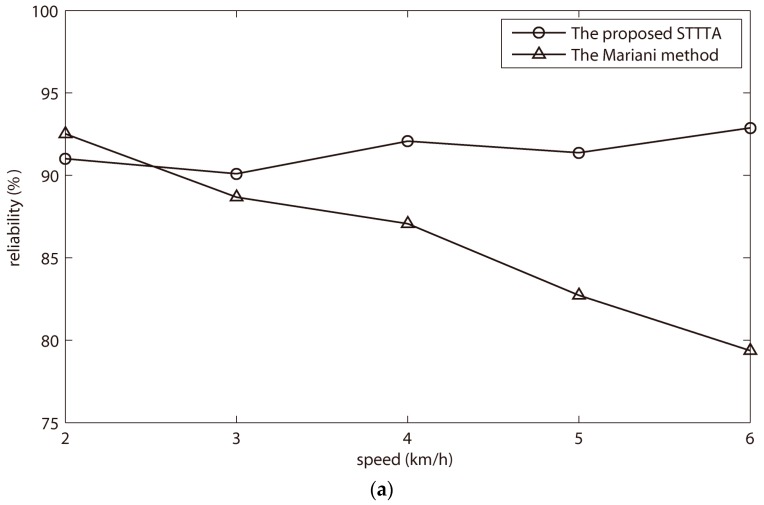

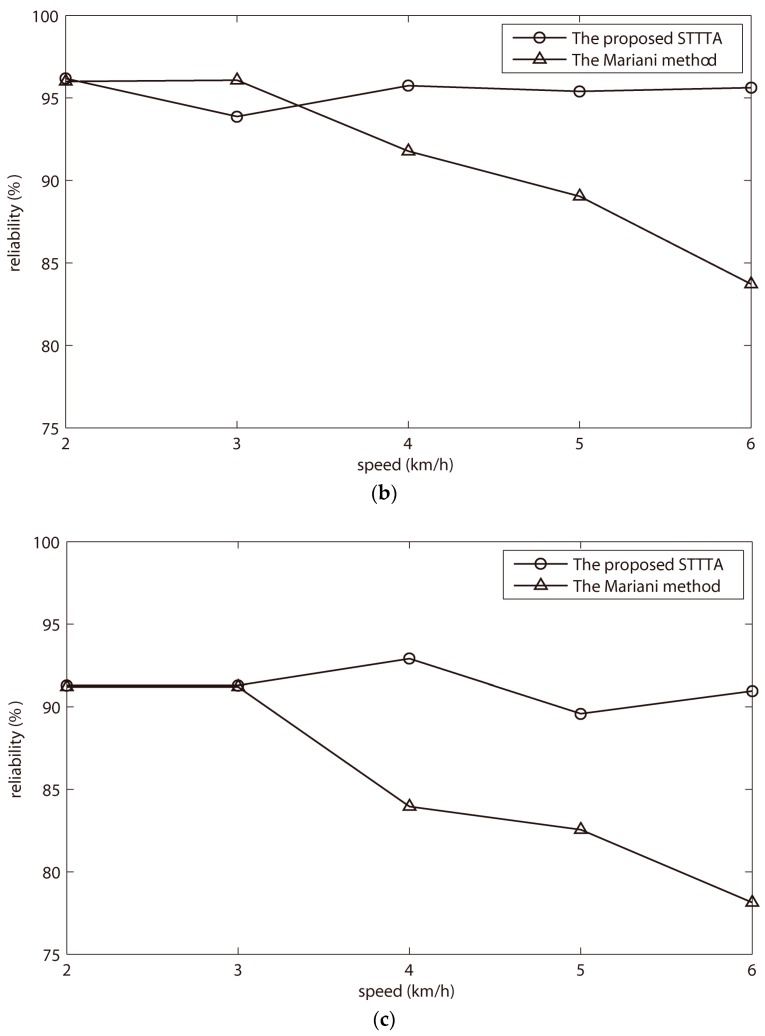

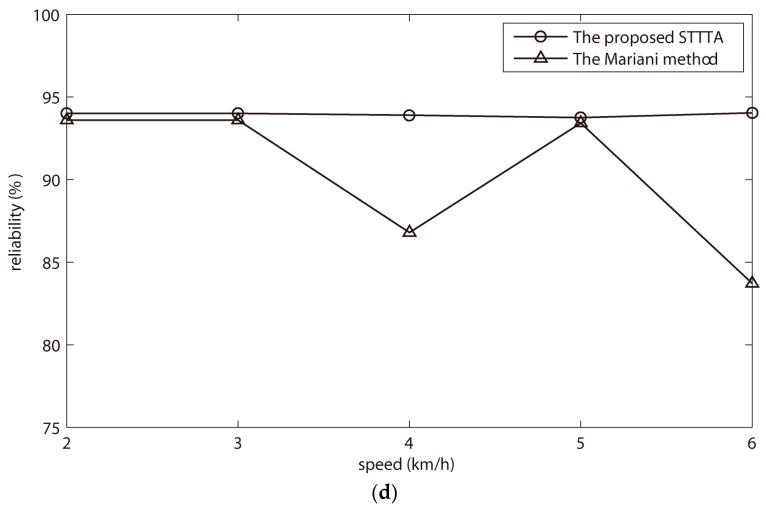
Reliabilities of the Mariani and STTTA method for one male subject at five walking speeds: (**a**) TAM method as reference method; (**b**) Lopez–Meyer method as reference method. Reliabilities of the Mariani and STTTA method for one female subject at five walking speeds; (**c**) TAM method as reference method; (**d**) Lopez–Meyer method as reference method.

To demonstrate the adaptability of the proposed STTTA, the experimental results of one male subject and one female subject were taken as examples. As shown in Figure 5a–d, the Mariani method could achieve high reliability at one walking speed, and then reduce significant reliability at other walking speeds. However, the proposed STTTA gained stable reliabilities at five walking speeds. The stability to speeds proved that the proposed STTTA was adaptable to different walking speeds.

## 4. Discussion

### 4.1. The Reason of Using Three Thresholds for GCFs Processing

**Figure 6:**
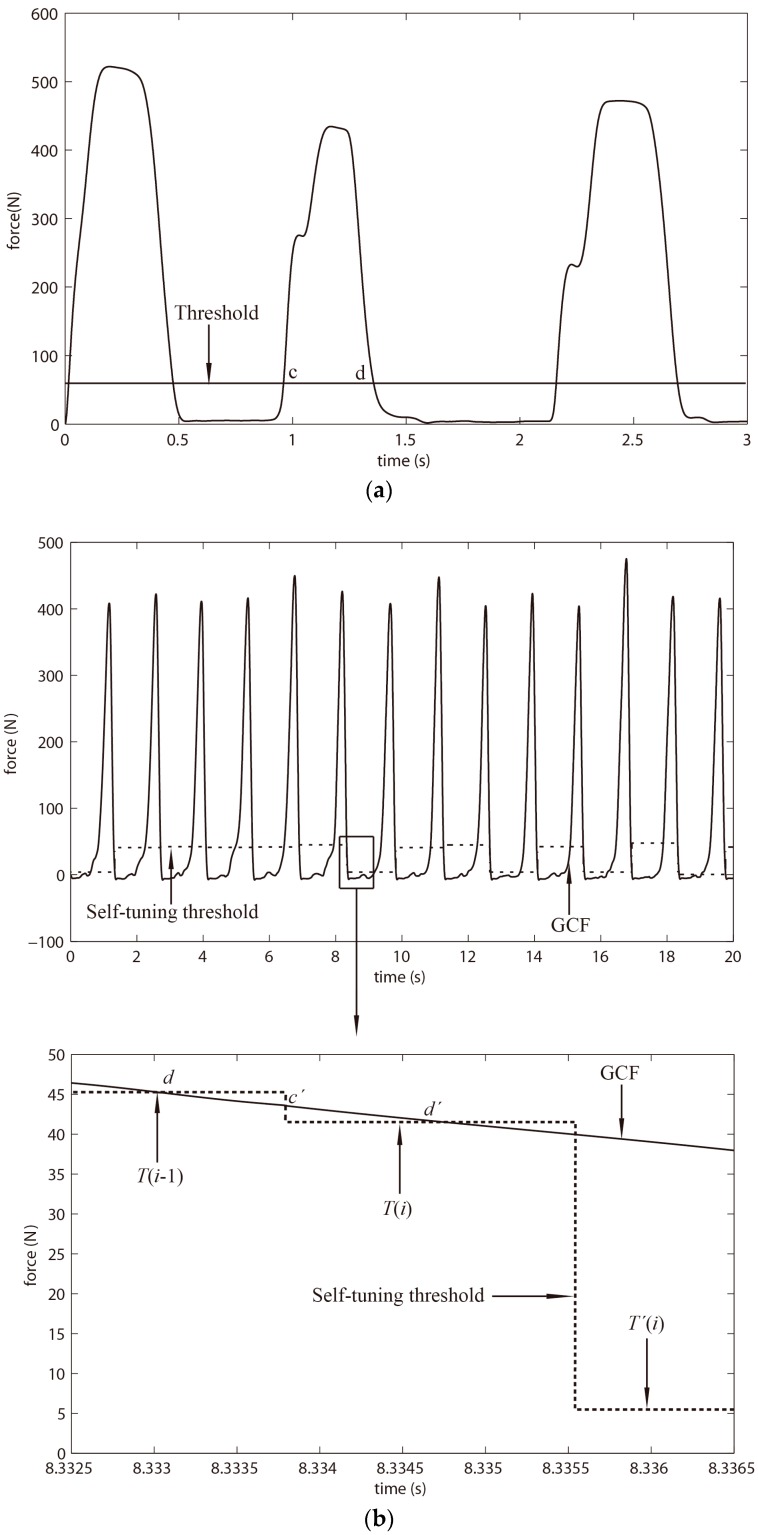
(**a**) the description of using one threshold for threshold adjustment; (**b**) demonstration of threshold adjustment using one threshold and analysis of twice threshold computations.

At the beginning, one threshold was expected to obtain the maximum and minimum GCFs of the present gait cycle, and the time point of a new threshold calculation for the next gait cycle. As shown in Figure 6a, the region (c,d) was used to search the maximum GCF of the present gait cycle, and the point d was used as the time point for new threshold calculation. However, the new threshold would be calculated twice (i.e., it should be only once) occasionally at one gait cycle as shown in Figure 6b. The threshold *T*(*i*), which was the correct threshold calculated for the next gait cycle at time point d. Nonetheless, it possibly existed that the GCFs after time point d were larger than the newly computed threshold *T*(*i*) in a new region (c’,d’). In this situation, the algorithm would search the maximum GCFs in region (c’,d’), and figured out an unexpected new threshold *T*’(*i*), which replaced the *T*(*i*) for threshold processing of the next gait cycle, at time point d’. Additionally, the secondly calculated threshold *T*’(*i*) was a false threshold for the next gait cycle. Similarly, using two thresholds also resulted in the same problem.

However, using three thresholds avoided the above-mentioned problem. As Figure 2 showed, the maximum and minimum GCFs were severally obtained in region (c, d) and (f, g); meanwhile, the new thresholds (i.e., *T_H_* and *T_L_*) were severally computed at time point e and h. The middle threshold T_M_ was to search the time point e and h, which avoided the new threshold computation at time point d or g. To sum up, the use of three thresholds avoided the problem of twice threshold computations.

### 4.2. Adaptability to Variable Speed Walking

Generally, when the subjects walk faster, their feet hit the ground harder, i.e., the magnitude of GCFs gets bigger. As a result, the magnitude of GCFs changes with the variation of walking speeds; meanwhile, the maximum GCF increases (or decreases) as the walking speed increases (or decreases). According to the formula in Equation (8), when the subjects walk slowly, the maximum GCF possesses small magnitude such that a small threshold is calculated. Similarly, when the subjects walk fast, the formula calculated a big threshold. As to variable speed walking, one computed threshold should be set for each walking speed to conduct correct gait phase detection. If the subject suddenly changes walking speed, the ground contact force changes accordingly. The subject will take one step, and the thresholds will be adjusted to the new speed because three thresholds of the next gait cycle are calculated by the GCFs of the present gait cycle. The proposed STTTA uses the maximum and minimum GCFs from the last gait cycle to calculate a threshold (i.e., *T_H_*) for the present gait cycle. The maximum and minimum GCFs of the last gait cycle are the closest to those of the present cycle. As a result, appropriate thresholds are calculated for each gait cycle, which correlates with speed variation. To sum up, the proposed STTTA is adaptable to variable speed walking.

### 4.3. Limitation of the Research

In our study, only healthy subjects have been studied on level ground, not taking the pathological subjects into account. The experiments were done on a treadmill because the method is not suitable for irregular terrain and stairs walking. Uneven road conditions and obstacles on the ground may lead to misdetections.

In the experiments, some rules of GPDA were not observed. However, the specific walking habits of individuals may employ the unused rules. The GPDA rules could be used to detect abnormal gaits for podiatric diagnoses.

## 5. Conclusions

This paper proposes a self-tuning triple-threshold algorithm that calculates adjustable thresholds to adapt to human walking. In addition, the adjustable thresholds are calculated in the walking process, and the same initial threshold values are set for all subjects in all experiments. For all subjects, high average reliabilities are gained when the proposed method is severally compared with two reference methods. For each subject, the proposed method acquires stable reliabilities in five experiments with different walking speeds each time. It comes to a conclusion that the proposed STTTA can be used to detect gait phases in real time, and shows adaptability to different walking conditions on level ground. In the future work, the method of gait phase detection would be studied on irregular terrains or when climbing stairs. Furthermore, it needs more types of sensors to detect the sub-phases of swing.

## Figures and Tables

**Table 1:** Rules of gait phase detection algorithm.

Heel	Ball	Gait Types	
on-ground	on-ground	Gait phase	Stance
off-ground	off-ground	Gait phase	Swing
on-ground	off-ground	Gait event	Heel-strike
off-ground	on-ground	Gait event	Heel-off

**Table 2:** Comparative reliability results for the proposed method.

Subjects	Gender	Mariani Method		The Proposed STTTA	
		Compared with TAM Method	Compared with Lopez–Meyer Method	Compared with TAM Method	Compared with Lopez–Meyer Method
1	Male	91.66%	80.70%	95.01%	87.50%
2	Male	86.80%	83.96%	93.07%	92.90%
3	Male	93.42%	82.55%	92.82%	89.58%
4	Male	83.72%	78.14%	94.14%	90.96%
5	Male	88.29%	82.28%	89.97%	80.00%
6	Male	91.16%	81.75%	96.49%	89.95%
7	Male	91.77%	84.40%	96.57%	91.07%
8	Male	93.93%	85.32%	92.68%	88.88%
9	Male	87.41%	77.79%	91.87%	94.58%
10	Male	88.33%	85.18%	88.49%	82.76%
11	Male	94.64%	84.12%	92.62%	88.00%
12	Male	93.00%	87.83%	96.54%	92.42%
13	Male	93.63%	84.47%	95.14%	87.45%
14	Male	94.03%	84.64%	96.03%	89.46%
15	Female	92.59%	86.21%	96.86%	92.58%
16	Female	95.92%	88.27%	95.5%	91.34%
17	Female	91.81%	88.42%	93.10%	91.58%
18	Female	89.81%	84.18%	86.00%	83.03%
19	Female	88.847%	79.80%	94.42%	88.92%
20	Female	84.47%	69.96%	94.02%	83.53%
21	Female	91.68%	90.84%	80.08%	93.95%
22	Female	81.07%	85.68%	85.09%	89.64%
23	Female	85.75%	90.54%	89.75%	94.39%
24	Female	80.56%	84.43%	88.86%	92.30%
Average	——	89.62%	83.81%	92.29%	89.45%
